# Litho- and biostratigraphic data of lower-middle Miocene sections in the Transylvanian basin and SE Carpathian Foredeep (Romania)

**DOI:** 10.1016/j.dib.2019.103904

**Published:** 2019-04-17

**Authors:** K. Sant, D.V. Palcu, E. Turco, A. Di Stefano, N. Baldassini, T. Kouwenhoven, K.F. Kuiper, W. Krijgsman

**Affiliations:** a Paleomagnetic Laboratory Fort Hoofddijk, Utrecht University, Utrecht, the Netherlands; b Department of Chemistry, Life Sciences and Environmental Sustainability, Parma University, Parma, Italy; c Department of Biological, Geological and Environmental Sciences, Catania University, Catania, Italy; d Department of Geosciences, Stratigraphy-Paleontology, Heidelberglaan 2, 3584 CS Utrecht, the Netherlands; e Department of Earth Sciences, Vrije Universiteit Amsterdam, the Netherlands

**Keywords:** Foraminifera, Calcareous nannofossils, Badenian flooding, *Streptochilus*, Paratethys

## Abstract

Litho- and biostratigraphic data are provided of 5 stratigraphic sections in Romania covering the “Badenian” marine flooding that occurred in the Central Paratethys during the middle Miocene (Langhian). The dataset includes stratigraphic logs and descriptions of the profiles, and biostratigraphic analyses on calcareous nannofossils and foraminifera. In addition, characteristic stratigraphic features and representative fossils, including tiny *Streptochilus* foraminifera in the Campiniţa section in the SE Carpathian Foredeep, are presented in photographs. The data show that the flooding is characterized by the sudden abundance of Langhian calcareous nannofossils and foraminifera with a strong Mediterranean affinity.

**Table:** Specifications table

Subject area	*Geology*
More specific subject area	*Stratigraphy, Micropaleontology, Field geology*
Type of data	*Table, images, figures, descriptive text*
How data was acquired	*Stratigraphic logging* *Microscope: Zeiss Axioscope (nannoplankton), Nikon SMZ stereomicroscope (benthic foraminifera), Zeiss stereomicroscope (planktonic foraminifera)* *SEM:. JEOL JXA-8530 F field emission electron probe analyzer (benthic foraminifera), Jeol 6400 (planktonic foraminifera)*
Data format	*Raw and analyzed.*
Experimental factors	*Collection of data was focused on determining the age and mode of the Langhian marine flooding in the Transylvanian Basin and SE Carpathian Foredeep by litho-biostratigraphic constraints.*
Experimental features	*Documenting the lithology and fossils (planktonic and benthic foraminifera, calcareous nannofossils, ostracods) in five semi-parallel stratigraphic sections.*
Data source location	*Transylvanian basin and SE Carpathian Foredeep, Romania (see text for GPS coordinates of all localities).*
Data accessibility	*Data are included in this article and supplemented Excel file.*
Related research article	*A. de Leeuw, S. Filipescu, L. Maţenco, W. Krijgsman, K. Kuiper, M. Stoica, Paleomagnetic and chronostratigraphic constraints on the Middle to Late Miocene evolution of the Transylvanian Basin (Romania): Implications for Central Paratethys stratigraphy and emplacement of the Tisza–Dacia plate, Glob. Planet. Change. 103 (2013) 82–98.*

**Table:** 

**Value of the data** • The calcareous nannofossil and planktonic foraminiferal bio-events from the study area can be compared to dated bio-events in the Mediterranean region in order to provide an age framework for the Paratethys region. • The litho-biostratigraphic data can be incorporated in future chronostratigraphic and paleoenvironmental studies in the Paratethys region. • The data provide the first report of tiny *Streptochilus/Bolivina* spp. foraminifera in the sections in the SE Carpathian Foredeep in Romania, allowing comparison to similar blooming events in other parts of the Paratethys Sea, Mediterranean and Atlantic Ocean.

## 1 Data

**Fig. 1:**
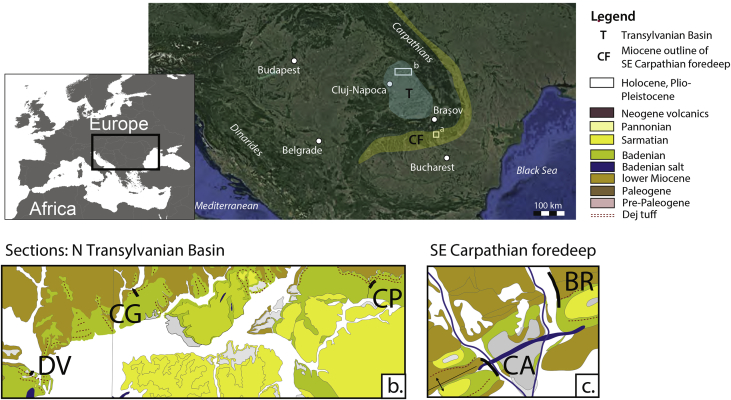
**Study area.** a) Position of the Transylvanian Basin, Carpathian Foredeep and study areas; b, c) sections marked on the geological map. Map modified after [16].

**Fig. 2:**
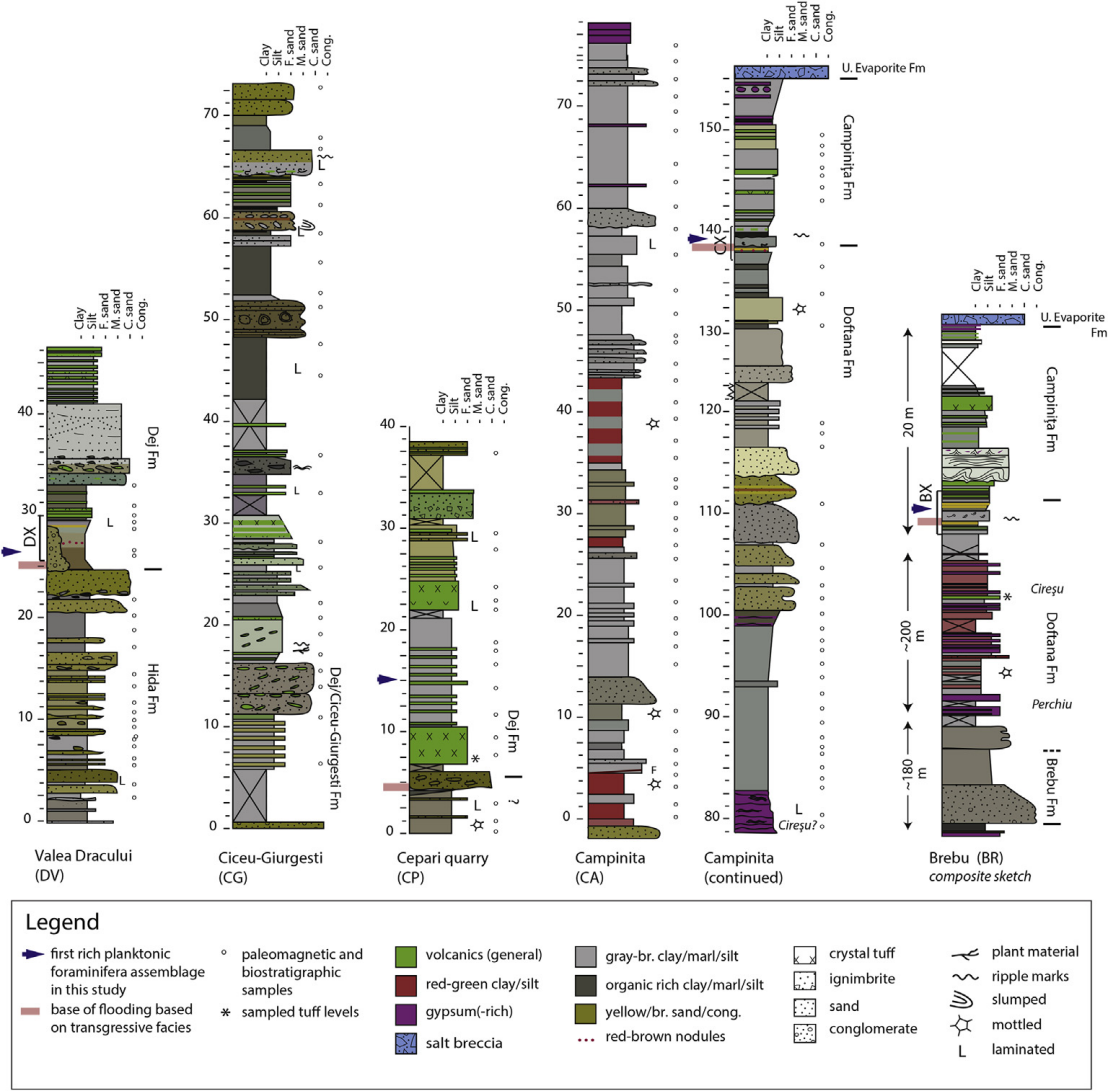
**Studied sections.** Lithology, formations and sample levels from five sections in the N Transylvanian Basin (DV, CG, CP) and SE Carpathian Foredeep (CA, BR). See legend for symbol explanation.

**Fig. 3:**
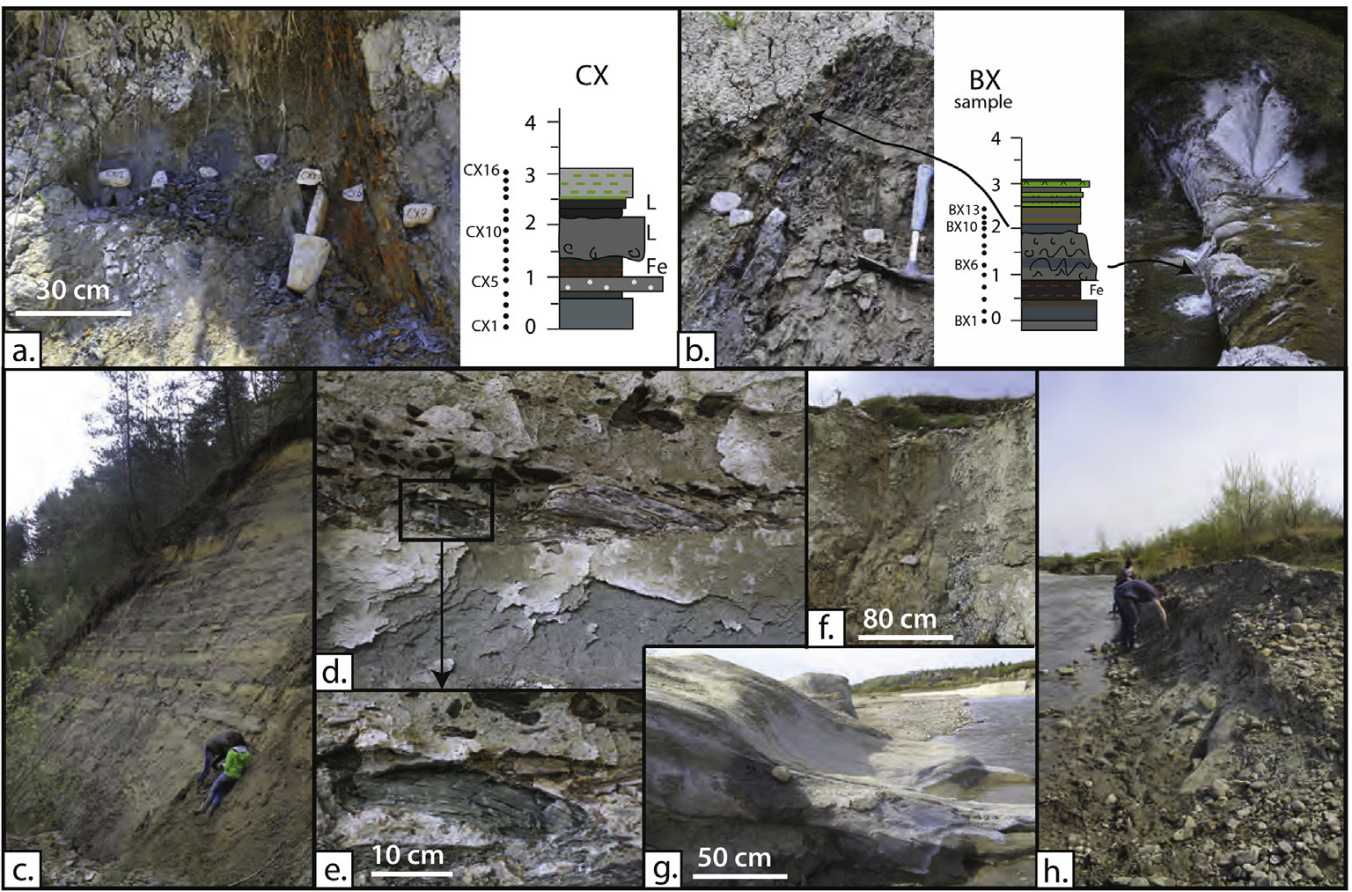
**Images of transgressive boundary sections.** a) CX: Campiniţa section, b) BX: Brebu section) and other characteristic intervals of the sections (c–h): c). Pre-flooding facies in Valea Dracului (DV), d,e). Intraclasts in sand channel in the top of DV. f, g, h) Characteristic elements of the Campiniţa river section: red layers (f), sand bodies (g) and gray silty clay (h).

The litho- and biostratigraphic data from the sections document a shift from restricted brackish-marine deposits to open marine deposition by the sudden appearance of abundant Mediterranean planktonic foraminifera (Fig. 1, Fig. 2). This transgressive interval was logged in detail in the Campiniţa and Brebu sections, located in the Carpathian bend area in the East Carpathian Foredeep, where both intervals share similar features (CX, BX; Fig. 2., Fig. 3a–c). In the SE Carpathian Foredeep, the onset of the open marine conditions is assigned to the planktonic foraminiferal zone MMi5b and calcareous nannofossil zones MNN4c-MNN5a. In the Transylvanian sections, the quality of the data is variable and the base of the flooding is in the range MMi4c to MMi5a (planktonic foraminifera) and MNN5a-MNN5b (calcareous nannofossils). The data can be used for a paleoenvironmental and chronostratigraphic interpretation of the research area.

## 2 Experimental design, materials, and methods

### 2.1 Sampling and analysis

The stratigraphic profiles were logged at ∼0.5–1 m resolution during a fieldwork campaign in May 2015. Biostratigraphic samples were taken with a resolution of 1–2 m from the Valea Dracului (DV), Ciceu-Giurgesţi (CG), Cepari (CP) and Campiniţa (CA) sections. Additionally, biostratigraphic samples were taken at an approximately 25 cm resolution in the intervals just below the marine transgression in Valea Dracului, Campiniţa and Brebu. The resolution is lower (4 m or more) in intervals with a lot of coarse-grained material, such as sands and volcanic material.

A total of 81 calcareous nannofossil smear slides were prepared for calcareous nannofossil analysis using standard methods [1], [2] and analyzed with a Zeiss Axioscope microscope under magnification 1000× at Catania University. According to the counting methodologies proposed by Refs. [2], [3], targeted counts of biostratigraphically significant taxa were performed, in order to obtain relative abundances. Specifically, 30 and 50 specimens within the genera *Sphenolithus* and *Helicosphaera* were counted, respectively.

A total of 88 samples for the analysis of foraminiferal assemblages were prepared partly at the Geolab of the Faculty of Geosciences of Utrecht University and partly at the Micropaleontology Laboratory of the University of Parma. Samples (about 250 g) were dried in an electric oven at 40 °C for approximately 2 days, were processed with H_2_O_2_ (3%), and washed over 63 and 125 μm sieves. A qualitative analysis of planktonic foraminiferal assemblages was carried out on the >125 μm fraction of the washed residues focusing on the biostratigraphic marker species and on the >63 μm fraction specifically for *Streptochilus* spp; representative taxa were picked and mounted on micropaleontological slides. The biostratigraphic scheme for the Mediterranean by Ref. [4] emended in Ref. [5] was adopted. The foraminiferal content of the samples is highly variable, from (nearly) absent to very abundant. The preservation is also variable, from very poor to good. The samples containing benthic foraminifera (>125 μm size fraction) were qualitatively examined and representative taxa were mounted on micropaleontology slides for reference.

### 2.2 Lithostratigraphy

#### 2.2.1 SE Carpathian Foredeep: Campiniţa and Brebu

The Miocene sediments in the SE Carpathian bend area were deposited in a former piggyback basin and subsequently incorporated into the Tarcău nappe. Similar deposits are found in the Râmnicu Vâlcea -Câmpulung – Câmpina – Slănic areas [6], [7]. In the study area, the Slănic tuff with *Globigerina* Marls that mark the Badenian flooding are known as the Campiniţa Formation [8].

The **Campiniţa** (CA; 45.136799°N, 25.711042°E) section is 155 m thick and located on the southern flank of an anticline along the western bank of the Prahova river. The outcrop covers the upper part of the Doftana, Campiniţa and Upper Evaporite Formations (Fig. 2). The lithology is dominated by brown- and green-gray clays and silts with occasional wavy bedded sandy levels. Continental mottled red and green clays mark the basal part (Fig. 3f. Thin gypsum levels appear from 60 m upwards. Around 80 m a prominent interval with wavy gypsum lamina intercalating with mm-scale organic rich shales is present, which likely represents the Cireşu gypsum [1] (Fig. 3). Thick yellow-gray fine to coarse mica-rich sand bodies stand out between 100 and 117 m (Fig. 3g. Some have current structures at their base and they often alternate with reddish marls in layers of 30–50 cm. Findings of vertebrate footprints and traces of raindrops suggest shallow water to intertidal conditions for part of the environment [9]. The base of the Campiniţa Fm is at 138 m (Fig. 1). The formation is dominated by an about 10 m-thick sequence of gray tuffaceous marls with three biotite-bearing tuffs (5–10 cm thick) with large middle Miocene planktonic foraminifera. The section is sealed by a sequence of sapropels, gypsum-rich conglomerates and salt breccia.

The 400 m-thick **Brebu** (BR; 45.185498°N, 25.775669°E) profile is found along a NE striking tributary of the Doftana river and covers almost the complete early Miocene succession including the uppermost Lower Gypsum, Cornu(?), Brebu and Doftana Formations (Fig. 2). After a basal interval of gypsum and black shales (about 50 m; Lower Evaporite Fm), the lower part of the stratigraphy is dominated by conglomerates and sands (Brebu Fm) gradually passing into microconglomerates and clays of the Doftana Fm. Sands of the Brebu Fm are sourced from the Perşani mountains, which currently form the internal part of the East Carpathians suggesting a low Carpathian topography at that time of deposition [6].

The upper part of the Brebu section is dominated by gray, green and red mottled clays and sandy silts including several gypsum layers (Doftana Fm). This part is poorly to non-exposed due to mudslides and vegetation, and was studied in more detail in the Campiniţa section. Two notable evaporitic intervals could be recognized; the Perchiu gypsum at the base of the Doftana Fm, and the Cireşu gypsum towards the top, serving as regional evaporitic marker levels (Fig. 3b). Another notable feature is a biotite-bearing tuff layer (20–50 cm thick) followed by a prominent gypsum bed. The middle Miocene Slănic tuff level is clearly visible in the top of the main Brebu profile. The transgressive boundary between the Doftana Fm and Campiniţa Fm is exposed in a parallel section towards the west next to the Brebu Manastirei cemetery (Fig. 3b). It can be summarized by about 15 m of tuffitic clays and tuffites. The Brebu section is topped by a layer of salt breccia.

The transgressive intervals in Campiniţa and Brebu sections both start with blue-gray clay and silt layers, followed by a dark sapropelic silt or clay interval full with orange-weathered iron oxides and barren in fossils. In both sections, this is followed by sands bearing water escape structures. The first rich planktonic foraminiferal assemblages appear within or just on top of these sand packages. In Brebu, the succession is followed by another resistant and prominent thick fine-to-medium green-white three-layered disturbed sand package with wavy lamination and iron coated ‘knobs’ at its base (Fig. 3b).

#### 2.2.2 NW Transylvanian Basin: Valea Dracului, Ciceu-Giurgesţi and Cepari

The Valea Dracului (DV), Ciceu-Giurgesţi (CG) and Cepari (CP) sections cover (parts of) the upper Hida Fm and Dej Fm in the NW Transylvanian basin (Fig. 1). In this region, several studies defined the NN4 nannofossil biozone (Burdigalian) for the whole exposed Hida Fm, and the NN5a biozone (Langhian; after [10]) for the Dej Fm [11], [12], [13].

The 46 m long **Valea Dracului** (DV) section (alternative names: Dej, Râpa Dracului) (47.147342°N, 23.859869°E) is exposed on the flanks of a river canyon and covers the upper Hida and Dej Formations (Fig. 1). The upper Hida Fm (0–24 m) displays gray brown clays with cm-scale sand and coaly lenses. These are occasionally perturbed by yellow fine to medium sand beds that pinch out laterally (Fig. 3d). At 20 m, thick coarse to medium sand beds with basal clay with rip up clasts and coal chips appear. The facies reflect a distal fan delta environment with some gravity flow associated deposits.

The Dej Fm begins with a wedging conglomerate lens (0–5 m thick) cutting laterally into a finer-grained succession with middle Miocene planktonic foraminifera. The basal part starts with orange fine sand grading first into slightly mottled brown-orange silt and later into gray brown silty clay. A horizontal layer of cm-scale iron nodules is present at the silt to clay transition. The brown clay is succeeded by distinct colored units: dark green bedded clay to sand, an orange clay level (1 cm), purple clay laminated with tuffites, gray marl with cm-lenses of tuffaceous sand, and green-white bedded tuffs (Fig. 1). This interval (24.3–28.7 m); DX in Fig. 2 is covered by massive volcanoclastics, towering high above the Dracului valley. The lowermost part is a tuffaceous sand with characteristic green elongated Dej tuff clasts with an erosive base, the middle part (∼6 m) is coarse sand with large scale cross beds, and the top is a bedded alternation of silts and greenish tuff layers (Fig. 3d and e). The Dej Fm was deposited on the shelf margin or in deeper marine settings. The prominent coarse tuffites/sands with basal rip-up clasts were interpreted as a submarine meandering channel reflecting high input of volcanic activity. This channel eroded into the underlying shelf deposits.

The 73 m thick **Ciceu-Giurgesti** section (CG; 47.241532°N, 24.032811°E) is exposed along a low-standing river gully [14]. Published a log and planktonic foraminiferal bio-events for the lower part of the Ciceu-Giurgesti section covering the early-middle Miocene boundary. During the fieldwork campaign in 2015, however, the lowermost part of the section presented by [23] was covered by sediment and vegetation, and could not be studied. Therefore, the here presented section starts just above the First Occurrence (FO) of *Praeorbulina glomerosa*, and thus covers the Dej Fm only. Here, the Dej Fm is also known as the Ciceu-Giurgeşti Fm, but this term is not used to avoid confusion with the other Transylvanian sections. The lowermost part of the profile (0–11 m) contains clays, silts and a thin conglomerate layer and is mostly unexposed (see [14] for details). Upwards a 5 m thick package of poorly sorted medium-coarse sand with elongated coarse greenish rip-up clasts and pebbles (<10 cm) stands out. The rip-up clasts are rich in tuffaceous material and occur in all sizes, the largest are 75 cm in length. The sand body is covered by an interval of silty clays and sands with (often) reworked green tuffs*,* and an ∼7 m interval dominated by volcanoclastic sands and cross-laminated layered tuffs. A tuff at ∼20 m stratigraphic height was dated at 14.38 ± 0.06 Ma [14]. The stratigraphy continues with dark gray clays intercalated with green tuff levels and an 1.5 m silty bed with algae mats and dark clay lenses. In the top of the CG section (>50 m) fine and medium grained sands with organic rich interbeds and some tuff intercalations stand out.

The 38 m thick **Cepari** section (CP; 47.242542°N, 24.425911°E) is well-exposed in a former quarry. Some authors infer a discontinuity at the base of the Langhian transgression in this region (∼7 km NW of Cepari) based on lithostratigraphy and microfaunal analysis [15]. In most places, the transgression begins with a conglomerate level followed by characteristic Langhian microfauna. The basal part (0–4.5 m) of Cepari section contains gray brown silty clay with occasional yellow sands, and may be part of the Hida Fm. It is overlain by two discontinuous beds of dark gray and gray clay, that are laterally cut by a maximally 3 m thick coarse-to-medium poorly sorted sand with sand intraclasts. The beginning of the Dej Fm is marked by the first 4 m thick tuff bed. Silty clays with tuffs including a second thick (3 m) tuff package are positioned on top. The section ends with tuffaceous yellow gray clays intercalating with some fine sands, and one thick volcanoclastic sand.

### 2.3 Biostratigraphic data

#### 2.3.1 Calcareous nannoplankton

**Fig. 4:**
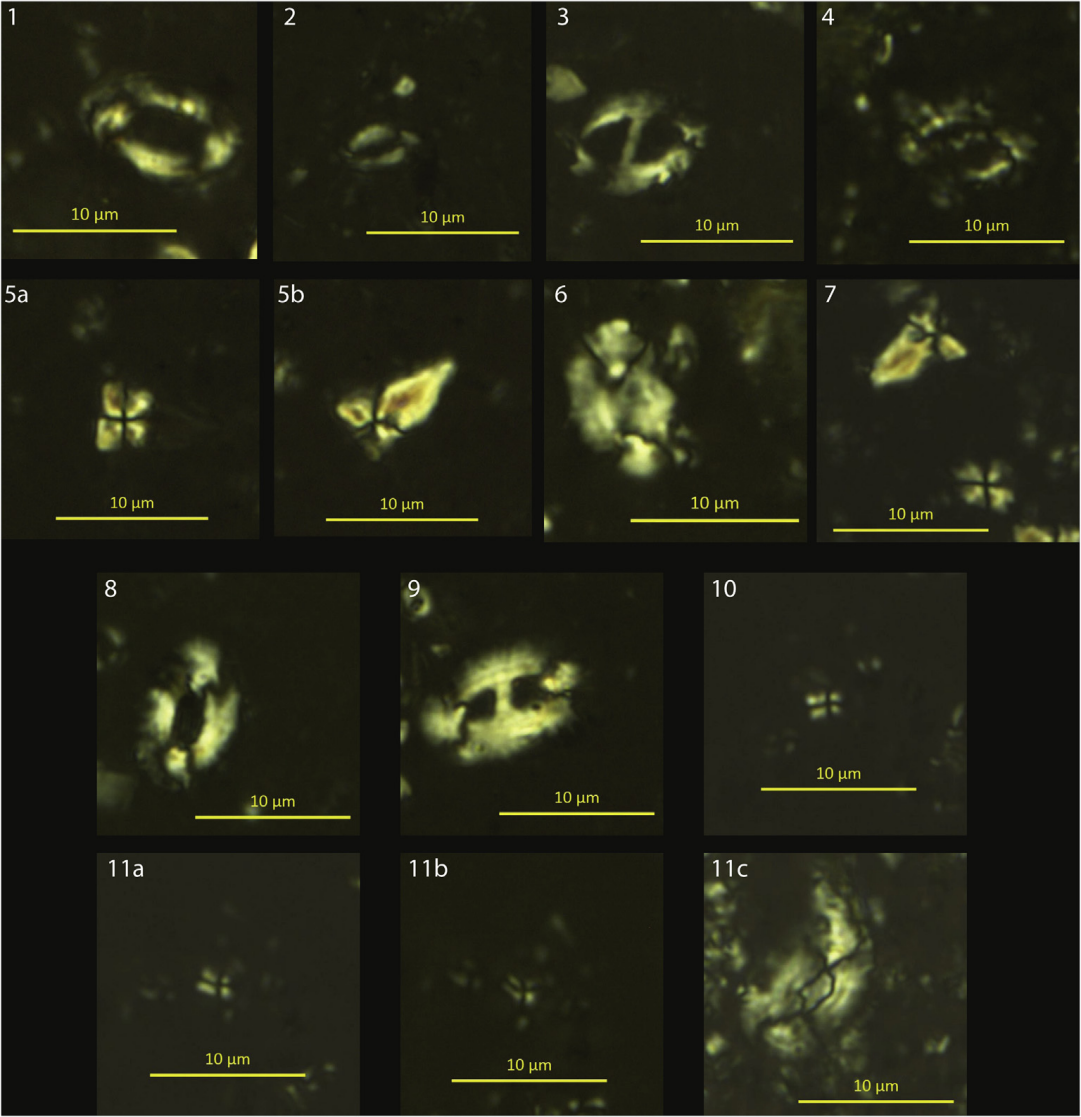
**Calcareous nannofossils**. (1) *Helicosphaera ampliaperta* (sample CA10); (2) *H. walbersdorfensis* (sample CG13); (3) *H. waltrans* (sample CA20); (4) *H. euphratis* (sample CX14); (5a, b) *Sphenolithus heteromorphus* (sample CG1) at 0° and 45°; (6) *S. heteromorphus* and *S. moriformis* (sample BX13); (7) *H. carteri* (sample BX13); (8) *H. intermedia* (sample CA9); (9) *H. ampliaperta* (sample CA19); (10) *H. mediterranea* (sample CA20); (11a, b, c) *S. disbelemnos* (sample CA55) at 0°, 20° and 40°.

In the Campiniţa (CA) section, calcareous nannofossils are common in nearly all of the analyzed samples and show a good to sometimes poor degree of preservation. The stratigraphically lower and intermediate samples (CA71 to CA09: 8.7–136,5 m) are dominated by middle to late Burdigalian assemblages from the MNN3b Zone to the MNN4a Subzone (Table 1, Fig. 7), with the exception of CA29, CA21 and CA20. The Burdigalian attribution is based on the recognition of *Sphenolithus heteromorphus* (Fig. 4) First Common Occurrence (it marks the base of the MNN4a Subzone), and on the continuous presence of *Helicosphaera ampliaperta* (Fig. 4) (its Last Common Occurrence defines the base of MNN4b Subzone). The analysis of samples CX3-CX16 (137.4–140.1 m) allows recognizing the early-middle Langhian MNN4c-MNN5a bio-chronostratigraphic interval. This attribution is based on the recognition of the *Sphenolithus heteromorphus* paracme interval in the lowermost sample (it defines the base of MNN4c Subzone), and by high abundances of the species in the following samples (the *S. heteromorphus* Paracme End defines the base of the MNN5a Zone). The calcareous nannofossil assemblages from samples CF16-1 (150.7 m) and CF12-1 (149.2 m), as well as samples CA01-CA07 (142,9–149.4 m), allowed depicting the middle to late Langhian MNN5b Subzone based on the recognition of poor percentages of *Helicosphaera walbersdorfensis* (Fig. 4) (its First Common Occurrence defines the base of the MNN5c Subzone) and the absence of *H. waltrans* (Fig. 4) (its Last Common Occurrence marks the base of MNN5b Subzone) (Fig. 4, Fig. 7).

**Table 1:** Qualitative biostratigraphic results and associated biozonations for calcareous nannofossils, planktonic and benthic foraminifera for all studied samples.

Campiniţa section		Calcareous nannofossils			Planktonic foraminifera	*marker species in bold*			Benthic foraminifera	Ostracods
Sample	Level (m)	Preservation	Main taxa	Biozone	Residue description	Main taxa	Biozone	Notes		
CF17	151.8				very fossiliferous, made up of planktonic foraminifera. Preservation good	assemblage is dominated by small-sized specimens of globorotalids		No markers		
CF16-1	150.7	good	*Sphenolithus moriformis (A), Helicosphaera carteri (A), H. mediterranea (C), H. eupratis (R), S. heteromorphus (R).*	MNN5b	very fossiliferous, made up of planktonic foraminifera. Preservation moderate (recrystallized and very often deformed)	assemblage is dominated by globigerinids		No markers		
CF14	150				terrigenous component < fossil content. Planktonic foraminiferal preservation moderate (recrystallized)	*Globigerinoides trilobus, Globorotalia praescitula*		No markers		
CA7	149.4	?	*H. carteri and S. heteromorphus abundant*	MNN5b	very fossiliferous, made up of planktonic foraminifera. Preservation good	assemblage of fraction >125μm is dominated by Orbulinids. *Orbulina suturalis, O. suturalis/universa transition, **O. universa***	MMi5b			
CF12-1	149.2	good	*Sphenolithus moriformis (C/A), S. heteromorphus (C), Helicosphaera carteri (A)*	MNN5b						
CF11.2	148.3	good	H. carteri and S. heteromorphus abundant	MNN5b	very fossiliferous, made up of planktonic foraminifera. Preservation good even if slightly oxidized, often deformed and fragmented	assemblage dominated by *Globigerinoides trilobus* (larger size) and small-sized specimens of globorotalids (*Globorotalia praescitula, Globorotalia* spp.)		No markers		
CA5	147.4	?	*H. carteri abundant, S. heteromorphus common; very few H. ampliaperta: Biozone MNN4b*	MNN4b (MNN5b if H. ampliaperta is reworked)	very fossiliferous, made up of planktonic foraminifera. Preservation good even if often deformed	*Dentoglobigerina* spp. (A), *Globigerinoides trilobus* (R), *Globorotalia* spp., *Praeorbulina glomerosa glomerosa*, *P. glomerosa circularis*, ***Orbulina suturalis***	MMi5a	Praeorbulina/Orbulina group common		
CA3	145.4	good	*H. carteri and S. heteromorphus abundant, H. walbersdorfensis present; C. premacintyrei, G. rotula common*	MNN5c	very fossiliferous, made up of planktonic foraminifera. Preservation good even if often deformed and fragmented	*Globigerinoides trilobus*, *Globigerinoides* cf *sicanus*, *Globorotalia praescitula*, *Globorotalia* spp.		No markers		
CF9	144.8				very fossiliferous, made up of planktonic foraminifera. Preservation good even if often deformed and sometimes fragmented	*Globigerinoides trilobus*, *Globigerinoides sicanus*, *Paragloborotalia siakensis* (sin.)(R), *Globorotalia praescitula*, *Globorotalia* spp., *Praeorbulina glomerosa circularis* (also evolute specimens), ***Orbulina suturalis***	MMi5a	Praeorbulina/Orbulina very rare and deformed		
CF8	143.5				very fossiliferous, made up of planktonic foraminifera. Preservation moderate (slightly recrystallized and often deformed)	*Globigerinoides trilobus*, *Globigerinoides* cf *sicanus*, *Paragloborotalia siakensis* (sin.)(R), *Globorotalia* spp., *Praeorbulina glomerosa circularis* (also evolute specimens), ***Orbulina suturalis***	MMi5a			
CA1	142.9	small-size	*H. carteri and S. heteromorphus abundant, very few H. walbersdorfensis*	MNN5b	very fossiliferous, made up of planktonic foraminifera. Preservation good even if often deformed	*Globigerinoides trilobus*, *Globigerinoides sicanus*, *Paragloborotalia siakensis* (sin.)(R), *Globorotalia* spp., *Praeorbulina glomerosa circularis* (also evolute specimens), ***Orbulina suturalis***	MMi5a	Praeorbulina/Orbulina gr rare		
CF7.2	141	good	H. carteri and S. heteromorphus abundant, very few H. walbersdorfensis; C. premacintyrei, G. rotula common	MNN5b	almost exclusively made up of undisaggregated sediment grains. Very diluted planktonic foraminiferal content. Small-sized specimens	*Globigerinoides trilobus*, *Paragloborotalia siakensis* (sin.), *Globorotalia praescitula*		No markers		
CX16	140.1	very good	*Helicosphaera carteri (A), H. ampliaperta (R), H. waltrans (R), Sphenolithus heteromorphus (C), S. moriformis (C)*	MNN5a	no terrigenous fraction, very fossiliferous, residue made up of planktonic foraminifera, preservation good.	*Paragloborotalia siakensis*, *Globorotalia scitula*, *Globigerinoides trilobus*, G. cf. *sicanus*, Praeorbulina glomerosa glomerosa, *P. glomerosa circularis*, ***Orbulina suturalis***	MMi5a			
CF6.2	140	good	H. carteri and S. heteromorphus abundant, few H. walbersdorfensis; C. premacintyrei, G. rotula common	MNN5b/c	very fossiliferous, made up of planktonic foraminifera. Preservation good even if sometimes deformed or fragmented	*Globigerinoides trilobus* (A), *Globigerinoides* cf. *sicanus*, *Paragloborotalia siakensis* (sin.), *Globorotalia praescitula*, *Praeorbulina glomerosa glomerosa*, *P. glomerosa circularis*, ***Orbulina suturalis***	MMi5a			
CX14	139.7	good	*Helicosphaera carteri (C/A), Sphenolithus heteromorphus (C/A), S. moriformis (C), H. walbersdorfensis (C), H. euphratis (R)*	MNN5c	very fossiliferous, no terrigenous fraction. Planktonic foraminifera represent almost the all residue, preservation moderate.	*Dentoglobigerina altispira* gr., *Globigerinoides trilobus*, *G. sicanus*, *Praeorbulina glomerosa curva*, *P. glomerosa glomerosa*, *P. glomerosa circularis*, ***Orbulina suturalis*** (apertures not always well visible)	MMi5a			
CF5.2	139.5	BARREN								
CX13	139.3	good	*Sphenolithus moriformis (C/A), S. heteromorphus (C), Helicosphaera carteri (A)*	MNN5b	inorganic fraction abundant, made up of oxydized and pyritized sediment fragments, pyrite. Plant remains abundant. Planktonic foramnifera common, moderately preserved, no benthic foraminifera	*Globigerina bulloides* gr., *Globigerinoides trilobus*, *Globoturborotalita woodi*, *Globorotalia scitula*, *Turborotalita* cf. *T. quinqueloba*, *Dentoglobigerina* spp., *G.* cf. *sicanus*, *Praeorbulina glomerosa curva*, *P. glomerosa glomerosa*, *P. glomerosa circularis*, ***Orbulina suturalis***	MMi5a			
CX12	139.3	good	*Helicosphaera carteri (C/A), Sphenolithus heteromorphus (C), S. moriformis (C), H. intermedia (R), H. waltrans (R), H. obliqua (R)*	MNN5a	very little residue; qz, mica. Planktonic foraminifera in trace, preservation moderate.	*Dentoglobigerina altispira* gr., *Turborotalita* cf. *T. quinqueloba*, *Globigerinoides trilobus*, ***Orbulina suturalis??*** (only 1 specimen, apertures not well visible)	MMi5a?			
CA8	138.7	very poor	UNDEFINABLE		inorganic fraction abundant (lithic grains), planktonic foraminifera in trace	rare *Streptochilus/Bolivina variabilis* in fraction <125 μm		no markers	miliolids common; rare and recrystallized hyaline species (Gavelinopsis? sp., Rosalina sp.)	abundant Loxoconcha punctatella
CX8	138.5	good	*Helicosphaera carteri (C/A), Sphenolithus heteromorphus (C), S. moriformis (C), H. waltrans (R), H. intermedia (R), H. ampliaperta (R), H. obliqua (R), H. mediterranea (R/C)*	MNN5a						
CX6	138.1	very poor	UNDEFINABLE		sediment fragments			barren		
CF3.2	138	very poor	S. moriformis, H. ampliaperta, D. variabilis and H. carteri are present	MNN4a?						
CX3	137.4	poor	*Helicosphaera carteri (C), Sphenolithus heteromorphus (RR), S. moriformis (C), H. mediterranea (R), H. ampliaperta (R)*	MNN4c	very little residue; sediment fragments, pyrite. Planktonic foraminifera rare, preservation good	*Globigerinoides trilobus, G.* cf. *sicanus, Dentoglobigerina altispira* gr., *Globorotalia praescitula*		No markers		
CX02	137.2				very little residue, mica (muscovite) very abundant, lithic grains rare			barren		
CX01	137				very little residue, mica (muscovite) very abundant, lithic grains rare, oxydized fragments rare, echinids remains, foraminifera in trace	small-sized globigerinids, *Turborotalita* cf. *T. quinqueloba*		Nearly barren, no markers		
CA9	136.5	good	*Helicosphaera carteri (C), Sphenolithus moriformis (C), H. euphratis (R), H. mediterranea (R), H. ampliaperta (C), H. walbersdorfensis (R)*	MNN3b	very little washed residue, muscovite abundant, pyritized fragments, foraminifera in trace, radiolarians.	One deformed planktonic foraminifer: *Globigerinoides sicanus??*; rare *Streptochilus/Bolivina variabilis* in fraction <125 μm. One *Morozovella* sp. (Paleogene)		nearly barren	rare benthic foraminifera: Bolivina plicatella, Bulimina elongata, miliolids.	
CF 2-1	136.5	very poor	UNDEFINABLE							
CF1.2	135.5	moderate	S. moriformis (C ), H. ampliaperta ( C), H. carteri (C ), H. euphratis (R )	MNN4a						
CA10	133.7	poor	*Helicosphaera carteri (C), Sphenolithus moriformis (C/A), H. intermedia (R), H. euphratis (R), H. mediterranea (R), H. ampliaperta (R), H. obliqua (R)*	MNN3b	very little residue, aggregated sediment, muscovite. Ostracods, radiolarians, plant remains. Foraminifera in trace	*Dentoglobigerina* spp., *Globigerina* spp.; rare *Streptochilus/Bolivina variabilis* in fraction <125 μm		nearly barren, no markers	benthic foraminifera extremely rare and recrystallized: Astrononion? sp., Cibicides lobatulus.	
CA11	130.9				inorganic fraction made up of abundant mica (mainly muscovite), common terrigenous fragments and quartz, foraminifera very rare	small-sized globigerinids, *Turborotalita* cf. *T. quinqueloba*; rare *Streptochilus/Bolivina variabilis* in fraction <125 μm		nearly barren, no markers		
CA12	118.9				inorganic fraction made up of only mica (muscovite), abundant plant remains	rare *Streptochilus/Bolivina variabilis* in fraction <125 μm		barren		
CA13	117.9	good	*Helicosphaera carteri (C), Sphenolithus moriformis (C), H. intermedia (R), H. euphratis (R), H. mediterranea (R), H. ampliaperta (R), H. walbersdorfensis (R)*	MNN3b	very little residue, muscovite very abundant, quartz, terrigenous fragments, some pyritized fragments, some plant remains. Foraminifera in trace, plant remains, radiolarians and spiculas	globigerinids, *Turborotalita* cf. *T. quinqueloba*		nearly barren, no markers	few benthic foraminifera: Bolivina spathulata, Bulimina elongata, B. striata, Cassidulina carinata, Cibicides sp., discorbids, Hanzawaia boueana, Oridorsalis umbonatus, Rosalina bradyi	
CA18	102.9				residue made up of mica, quartz, lithic grains, terrigenous fragments. Very diluted foraminiferal (planktonic and benthic) content. Radiolarians??	globigerinids		no markers	fragmented benthic foraminifera: fragments of nodosarids, Ammonia, agglutinated taxa	
CA19	101.7	good	*Helicosphaera carteri (C), Sphenolithus moriformis (C/A), H. intermedia (R), H. euphratis (R), H. mediterranea (R), H. ampliaperta (C), H. walbersdorfensis (R)*	MNN3b	very little residue, terrigenous fragments abundant, lithic grains, rare oxidized fragments, planktonic foraminifera very rare and poorly preserved, possible reworking	*Paragloborotalia siakensis*, *Globigerinoides trilobus, G.* cf. *sicanus* (apertures not visible), ***Praeorbulina glomerosa glomerosa*****1 spec. !**	MMi4c?	This datum and thus the subzonal attribution is very weak		
CA20	100.2	good	*Helicosphaera carteri (C), Sphenolithus moriformis (C/A), S. heteromorphus (C), H. euphratis (R/C), H. mediterranea (R/C), H. ampliaperta (R), H. intermedia (R), H. scissura (R), H. obliqua (R), H. recta (R), H. waltrans(R)*	MNN5a	very little residue, muscovite abundant, lithic grains, quartz, rare pyritized fragments	Just 1 planktonic foraminifer: *Globigerinoides sicanus* (3 apertures)		nearly barren		
CA21	98.7	good	*Helicosphaera carteri (C/A), Sphenolithus moriformis (A), H. euphratis (C), H. ampliaperta (C), H. intermedia (R), S. heteromorphus (C), H. obliqua (R), H. mediterranea (R/C)*	MNN4a	very little residue, mica very abundant, lithic grains terrigenous fragments, small-sized foraminifera in trace			nearly barren, no markers		
CA22	97.5	poor	*Helicosphaera carteri, H. euphratis, H. scissura, H. waltrans, Sphenolithus hetromorphus, S. moriformis*	MNN5a	very little washed residue (>63μm)			barren		
CA23	96.5				very little residue, lithic grains, qz, mica			barren		
CA24	95.4				very little residue, lthic grains, qz, mica, plant remains			barren		
CA25	93.9				residue made up of mica (mainly muscovite), qz, rare lithic grains, terrigenous fragments. Very diluted foraminiferal (planktonic and benthic) content. Radiolarians?	globigerinids		no markers	rare and fragmented benthic foramininifera: fragments of nodosarids, Ammonia, Elphidium, Bulimina, Uvigerina spp.; agglutinated taxa?	
CA26	92.4				residue made up of qz, mica (mainly muscovite), lithic grains. Planktonic foraminifera in trace (some reworked)	*Globigerinoides trilobus*, *Globigerina praebulloides*		nearly barren, no markers	rare calcareous benthic foraminifera (Ammonia sp., Bulimina sp., fragments of nodosarids)	
CA27	90.9				very little residue, terrigenous and lithic grains, pyritized fragments, mica, qz			barren		
CA28	89.5				residue made up of mica (mainly muscovite), qz, rare lithic grains. Very diluted planktonic foraminiferal content	*Globigerina praebulloides*, *Globigerinella* spp.		No markers		
CA29	88	good	*Helicosphaera carteri (C/A), Sphenolithus moriformis (C/A), S. heteromorphus (R), H. euphratis (R/C), H. mediterranea (R/C), H. ampliaperta (R), H. scissura (R), H. obliqua (R), H. recta (R), H. walbersdorfensis (R)*	MNN4c	very little residue, lithic grains, qz, rare pyrityzed fragments, rare plant remains. Planktonic foraminifera in trace reworked (Cretaceous?)			barren		
CA30	87				very little residue, terrigenous fragments, lithic grains, qz, muscovite, plant remains. Planktonic foraminifera in trace reworked (Cretaceous?)			nearly barren	rare fragments of agglutinated benthic foraminifera	
CA33	83.9				little residue, lithic grains, qz, muscovite, rare plant remains. Planktonic foraminifera absent			barren		
CA34	82.9				very little residue, lithic grains, mica (muscovite) (A), oxydized fragments, lignite and plant remains. Planktonic foraminifera absent.			barren		
CA35	80.4				terrigenous grains, common transparent crystals (gypsum?). Planktonic foraminifera absent.			nearly barren	rare fragments of agglutinated benthic foraminifera	
CA36A	79.4	good	*Helicosphaera carteri (C), Sphenolithus moriformis (C/A), S. heteromorphus (R), H. intermedia (R/C), H. euphratis (R/C), H. mediterranea (R/C), H. ampliaperta (C), H. scissura (R), H. obliqua (R)*	MNN4a	very little residue, lithic grains, qz, rare plant remains			barren		
CA40B	71.1				muscovite and aggregated sediment			barren		
CA44	62.6				little residue, lignite (A), oxydized and pyritized terrigenous grains, mica, quartz. Planktonic foraminifera in trace	*Globigerinoides trilobus* (4 specimens), *Globorotalia* sp. (1 specimen), *Globoturborotalita woodi* (1 specimen), ***Praeorbulina glomerosa glomerosa*****(1 specimen)**	MMi4c?	Nearly barren: 1 specimen of Praeorbulina. This datum and thus the subzonal attribution is very weak	rare fragments of benthic foraminifera	
CA45	60.3				very little residue, terrigenous grains, oxydized and pyritized fragments			barren		
CA46B	55.3	good	*Helicosphaera carteri (C), Sphenolithus moriformis (C/A), S. heteromorphus (R), H. intermedia (R/C), H. euphratis (R), H. mediterranea (R/C), H. ampliaperta (C), H. obliqua (R)*	MNN4a	very little residue, qz, terrigenous fragments, lithic grains, mica, planktonic foraminifera in trace.	*Globigerinoides trilobus* (2 specimens), *Praeorbulina* sp. very badly preserved (1 specimen)		nearly barren	rare fragments of (mainly agglutinated) benthic foraminifera	
CA47	51.8	good	*Helicosphaera carteri (C), Sphenolithus moriformis (C/A), S. heteromorphus(R), H. intermedia (R), H. euphratis (R), H. mediterranea (R/C), H. ampliaperta (C), H. recta (R)*	MNN4a						
CA55	37.3	good	*Helicosphaera carteri (C), Sphenolithus moriformis (C/A), S. disbelemnos (R), H. intermedia (R), H. euphratis (R), H. mediterranea (R/C), H. ampliaperta (R/C)*	MNN3b	very little residue, terrigenous fragments, lithic grains, muscovite abundant, rare plant remains, very rare planktonic foraminifera (poorly preserved).	*Globigerinoides trilobus, G. sicanus* (elongated and less elongated morphotypes, maybe 1 with 3 apertures) *Paragloborotalia* cf. *siakensis*				
CA60	29.6	poor	*Helicosphaera carteri (C), Sphenolithus moriformis (C), H. intermedia (R), H. euphratis (R), H. mediterranea (R), H. ampliaperta (R)*	MNN3b	very little residue, quartz, lithic grains, muscovite, rare plant remains, plantonic foraminifera in trace	*Globigerinoides trilobus* (1 specimen), *Dentoglobigerina* sp.		nearly barren	rare fragments of benthic foraminifera	
CA71	8.7	poor	*Helicosphaera carteri (C), Sphenolithus moriformis (C), H. euphratis (R), H. mediterranea (R), H. perch-nielseniae(R), H. scissura (R), H. ampliaperta (R), H. recta (R)*	MNN3b	very little residue, rare muscovite			barren		

Ciceu-Giurgeşti Section		Calcareous nannofossils			Planktonic foraminifera	*marker species in bold*			Benthic foraminifera	Ostracods
Sample	Level (m)	Preservation	Main Taxa	Biozone	Residue description	Main taxa	Biozone	Notes		
CG24	∼80	good	*Helicosphaera carteri (A), S. moriformis (A), H. walbersdorfensis (R)*	MNN5c	quartz (A), lithic grains, sediment fragments. Foraminifera in trace (benthic > planktonic)			nearly barren, no markers	rare fragments of benthic foraminifera	
CG23	72.8				sediment fragments, quartz, rare biotite, lithic grains, plant remains.			nearly barren, no markers		
CG22	67.8	good	*Helicosphaera carteri (C/A), Sphenolithus heteromorphus (RR), S. moriformis (C/A), H. mediterranea (R)*	MNN5b	sediment fragments, quartz, rare biotite, lithic grains, plant remains.			nearly barren, no markers		
CG18	57.9	very poor	UNDEFINABLE							
CG17	55.4	good	*Helicosphaera carteri (C/A), Sphenolithus heteromorphus (C), S. moriformis (C/A), H. vedderi (R)*	MNN5b	inorganic fraction abundant (sediment fragments). Echinid remains, ostracods. Foraminifera common, moderately/poorly preserved, almost exclusively benthic. Planktonic foraminifera in trace	*Globigerinoides trilobus* (1 specimen), *Globigerina praebulloides* (1 specimen)		no markers	relatively rich. Ammonia tepida, Bolivina spathulata, Bulimina elongata, Cibicides dutemplei, C. ungerianus, Discanomalina coronata, discorbids/glabratellids, Elphidium spp., Fursenkoina acuta, Globobulimina sp., Nonion sp., Rosalina bradyi, large Uvigerina spp. (U. cf. acuminata, U. continuosa, U. semiornata). Few but large agglutinated taxa, a.o. textularids. Large, reworked miliolids.	*Citheridea acuminata*
CG16	52.4	poor	*Helicosphaera carteri (C), Sphenolithus heteromorphus (C/R), S. moriformis (C/R), H. vedderi (R)*	MNN5b						
CG14	47.5	poor	*Helicosphaera carteri (C), Sphenolithus heteromorphus (C), S. moriformis (C), H. walbersdorfensis (C), H. intermedia (R),H. mediterranea (R), H. vedderi (R), Discoaster musicus (C)*	MNN5c	inorganic fraction prevalent (lithic grains, sediment fragments, quartz, glauconite). Planktonic foraminifera rare, moderately/poorly preserved. Benthic foraminifera more abundant.	*Globigerina praebulloides*, *Globigerinoides trilobus*, ***Orbulina suturalis*** (some specimens are more evoluted toward *O. universa*)	MMi5a		relatively rich. Bolivina spathulata, Bulimina elongata, Cibicides dutemplei, C. ungerianus group (C. cf. pachyderma, C. ungerianus, C. cf. ungerianus), discorbids, Elphidium sp., Globocassidulina subglobosa, nodosariids, Pullenia bulloides, Spaeroidina bulloides, large Uvigerina spp. (U. continuosa, U. semiornata, U. venusta). Few but large agglutinated taxa, a.o. Spiroplectinella carinata.	
CG13	44.5	poor	*Helicosphaera carteri (C), Sphenolithus heteromorphus (C), S. moriformis (C/R), H. walbersdorfensis (C), H. euphratis (R)*	MNN5c	sediment fragments dominant, quartz, lithic grains, mica. Foraminifera in trace: few benthic, few planktonic foraminifera			nearly barren, no markers	Very few benthic foraminifera (Uvigerina, Bulimina)	
CG12	36.4	poor	*Helicosphaera carteri (C), Sphenolithus heteromorphus (C/R), S. moriformis (C), H. walbersdorfensis (C), H. vedderi (R)*	MNN5c	inorganic fraction subordinate to organic one. Foraminifera abundant, moderately preserved but very often deformed.	*Globigerina praebulloides* (A), *Globigerinella pseudobesa*, *Globorotalia scitula*, ***Orbulina suturalis*** (1 specimen)	MMi5a			
CG11	33	good	*Helicosphaera carteri (C), Sphenolithus heteromorphus (C), S. moriformis (C/A), H. intermedia (R)*	MNN5b	inorganic fraction prevalent (sediment fragments, quartz, rare pyritized fragments). Planktonic foraminifera rare, moderately/poorly preserved (often deformed).	*Globigerinoides trilobus* (A), ***Praeorbulina glomerosa circularis***, ***Orbulina suturalis*** (often deformed)	MMi5a	Praeorbulina and Orbulina poorly preserved (often deformed and apertures not well visible).		
CG10	29	poor	*Helicosphaera carteri (C), Sphenolithus heteromorphus (C), S. moriformis (C), H. intermedia (R), H. mediterranea (R)*	MNN5b	sediment fragments dominant, quartz, rare pyritized fragments, plant remains. Foraminifera in trace	*Globigerinoides trilobus, G.* cf. sicanus		nearly barren, no markers		
CG8	22.1	good	*Helicosphaera carteri (C), Sphenolithus heteromorphus (C), S. moriformis (C), H. intermedia (R), H. mediterranea (R)*	MNN5b	inorganic fraction dominant (sediment fragments, quartz, rare pyritized fragments). Planktonic foraminifera common, moderately/poorly preserved (often deformed).	*Globigerinoides trilobus* (A), *G.* cf. *sicanus*, ***Praeorbulina glomerosa circularis*** (some specimens evoluted toward *O. suturalis*)	MMi4d		Rare, fragmented benthic foraminifera	
CG5	17.1	good	*Helicosphaera carteri (C), Sphenolithus heteromorphus (C), S. moriformis (C), H. intermedia (R), H. mediterranea (R)*	MNN5b						
CG2	8.8									
CG1	6.5	good	*Helicosphaera carteri (C/A), Sphenolithus heteromorphus (C/A), S. moriformis (C), H. obliqua (R), H. mediterranea (R), H. euphratis(R)*	MNN5b	inorganic fraction dominant (sediment fragments, quartz). Planktonic foraminifera common, poorly preserved (often deformed, sometime pyritized). No benthic foraminifera	*Globigerinoides trilobus* (A), *Dentoglobigerina* spp., *G. sicanus*, ***Praeorbulina*****cf.*****glomerosa glomerosa ?*** (1 specimen).	MMi4c?	Preservation of Praeorbulina specimens is very poor (recrystallized and deformed). Uncertain interpretation.		

Valea Dracului section		Calcareous nannofossils			Planktonic foraminifera	*marker species in bold*			Benthic foraminifera
Sample	Level (m)	Preservation	Main taxa	Biozone	Residue description	Main taxa	Biozone	Notes	
DV22	33	good	*Helicosphaera carteri (C), Sphenolithus heteromorphus (C), S. moriformis (C)*	MNN5b	inorganic fraction subordinate to organic one, planktonic foraminifera abundant and moderately preserved (often deformed)	*Dentoglobigerina* abundant, *Praeorbulina glomerosa glomerosa*, ***Orbulina suturalis*** (some specimens are evoluted toward *O. universa*)	MMi5a	O. suturalis: a few specimens seem very close to O. universa but preservation is not optimal. Probably very close to MMI5b.	
DV21	30.7	good	*Helicosphaera carteri (A), Sphenolithus heteromorphus (C), S. moriformis (C), H. waltrans(R), H. mediterranea (R), H. intermedia (R)*	MNN5a	inorganic fraction subordinate to organic one, planktonic foraminifera abundant and moderately preserved (sometimes flattened)	*Dentoglobigerina* abundant, *Globigernoides trilobus, G.* cf. *sicanus*, ***Praeorbulina glomerosa glomerosa***, ***Praeorbulina glomerosa circularis*** (some specimens are evoluted toward *Orbulina suturalis*)	MMi4d		
DV20	30	good	*Helicosphaera carteri (C), Sphenolithus heteromorphus (C), S. moriformis (C), H. waltrans(R), H. mediterranea (R), Discoaster variabilis (C)*	MNN5a	terrigenous grains, planktonic foraminifera common and poorly preserved (sometimes flattened)	*Globigerinoides trilobus/sicanus*, ***Praeorbulina glomerosa curva***, ***Praeorbulina glomerosa circularis***	MMi4d		
DV19	29.5				inorganic fraction dominant (terrigenous grains, quartz, mica). Fish scales. Sediment sorting. Planktonic foraminifera rare to common, preservation poor	*Dentoglobigerina* spp., *Globigerinoides trilobus*, *Globorotalia praescitula*, *Praeorbulina* spp. (deformed)		No markers	rare benthic foraminifera, reworked? Cibicides lobatulus (1 specimen), C. cf. ungerianus?. Fragments of agglutinated species (?Textularia sp.)
DV18	28	poorly	*S. moriformis and H. carteri are present*	MNN4c?					
DV19d	26.5	poor	*Helicosphaera carteri (C), Sphenolithus heteromorphus (C), S. moriformis (C), H. ampliaperta (R), H. waltrans(R), H. mediterranea (R), H. intermedia (R), H. scissura (R)*	MNN5a	quartz and lithic fragments abundant			barren	
DV19c	26.3	BARREN							
DV19b	26.1	very poor	UNDEFINABLE		quartz and lithic fragments			barren	
DV19a	25.7	good	*Helicosphaera carteri (C), Sphenolithus heteromorphus (C), S. moriformis (C), H.mediterranea (R), H. ampliaperta(R)*	MNN5a				barren	benthic foraminifera very rare. A miliolid, Elphidium sp., Textularia sp. (each 1 specimen)
DV17	21.8	BARREN			little residue, quartz, lithic grains, oxidized fragments, no benthic foraminifera, planktonic foraminifera in trace	just 2 specimens: *Dentoglobigerina* sp., ***Praeorbulina glomerosa glomerosa*** (evoluted specimens toward circularis)	MMi4c?	nearly barren; based on only a single occurrence	
DV16	20.3	BARREN			quartz abundant, lithic grains abundant, not well sorted			barren	
DV14	17	BARREN			quartz abundant, lithic grains abundant, well sorted	*Globigerinoides trilobus*		nearly barren, no markers	
DV13	14.3	BARREN			residue made up of sediment fragments, quartz, mica (muscovite and biotite), rare oxydized and pyritized fragments, plant remains			barren	
DV12	12.9	BARREN			residue made up of sediment fragments, quartz, mica (muscovite and biotite), rare oxydized and pyritized fragments, plant remains			barren	
DV11	11.6	BARREN			residue made up of sediment fragments, quartz, mica (muscovite and biotite), rare oxydized and pyritized fragments, plant remains			barren	
DV03	5.6	BARREN			residue made up of sediment fragments, quartz, mica (muscovite and biotite), rare oxydized and pyritized fragments			barren	

Cepari Section		Calcareous nannofossils			Planktonic foraminifera	*marker species in bold*			Benthic foraminifera	Ostracods
Sample	Level (m)	Preservation	Main Taxa	Biozone	Residue description (>125 μm)	Main taxa	Biozone	Notes		
CP10	18.7	good	*Helicosphaera carteri (C), Sphenolithus heteromorphus (C), S. moriformis (C), H. intermedia (R), H. euphratis(R)*	MNN5a	inorganic fraction common, planktonic foraminifera abundant but very poorly preserved (very often deformed)	***Praeorbulina glomerosa glomerosa***, ***Praeorbulina glomerosa circularis, O.*****cf.*****suturalis***, *Paragloborotalia siakensis* rare	MMi4d	Praeorbulina very poorly preserved (recrystallized and deformed)		
CP9	16.7	?		MNN5a						
CP7B	14.7	good	*Helicosphaera carteri (C), Sphenolithus heteromorphus (C), S. moriformis (C), H. vedderi (R)*	MNN5a	inorganic fraction rare, planktonic foraminifera very abundant but very often deformed	*Globigerinoides trilobus, G.* cf. *sicanus*, ***Praeorbulina glomerosa curva***, ***Praeorbulina glomerosa glomerosa***, *Paragloborotalia siakensis* rare	MMi4c	Praeorbulina very poorly preserved (recrystallized and deformed)		
CP6	11.7	?		MNN5b						
CP5	9.2	poor		MNN5b?						
CP4	7.7	?		MNN5b						
CP3B	3.4	BARREN			terrigenous fraction abundant (quartz, lithic grains, rare mica), planktonic foraminifera common poorly preserved (often deformed)	*Globigerinoides trilobus, G.* cf. *sicanus*, *Paragloborotalia* cf. *siakensis* trace		no markers		
CP3A	3.4	BARREN			dirty, terrigenous material			barren		
CP2	1.4	BARREN			pyritized terrigenous fraction, pyritized burrows abundant, echinids remains, planktonic foraminifera in trace	*Globigerina bulloides*, *Globigerinoides trilobus*		no markers	benthic foraminifera relatively abundant. Ammonia beccarii and A. tepida, Bulimina elongata, many Cassidulina carinata, few cibicidids (a.o. C. lobatulus), Discanomalina coronata, Elphidium spp., Fursenkoina acuta, Gavelinopsis lobatula, Globobulimina spp., Porosononion granosum. Few large agglutinated taxa: Spiroplectinella deperdita, Textularia spp. Large reworked miliolids.	Cytheridea acuminata, Loxoconcha kochi, Pterygocythereis calcarata
CP1	0.1	BARREN								

Brebu section		Calcareous nannofossils			Planktonic foraminifera	*marker species in bold*		
Sample	Level (m)	Preservation	Main taxa	Biozone	Residue description (>125 microm)	Main taxa	Biozone	Notes
BX13	2.4	good	*Helicosphaera carteri (C), Sphenolithus heteromorphus (C), S. moriformis (C), H.intermedia (R), H. euphratis(R)*	MNN5b	very fossiliferous, no terrigenous fraction, only rare pyritized fragments and burrows. Preservation of planktonic foraminifera moderate (recrystallized and sometimes deformed)	*Globigerinoides trilobus*, *Dentoglobigerina* spp., *Praeorbulina* abundant but the apertures are not always visible, *P.* cf. *glomerosa curva*, *P. glomerosa glomerosa*, *P. glomerosa circularis*, ***Orbulina suturalis***	MMi5a	A few specimens seem to be transitional to O. universa.
BX10	2	good	*Helicosphaera carteri (A), Sphenolithus heteromorphus (C), S. moriformis (C), H.ampliaperta (R), H. waltrans, H. intermedia (R)*	MNN5a	very little washed residue; inorganic fraction rare (sediment fragments, pyritized fragments, quartz, mica). Planktonic foraminifera abundant, preservation moderate/poor (recrystallized, deformed)	*Globigerinoides trilobus, G.* cf. *sicanus*, *Globorotalia praescitula*, *Globoturborotalita woodi*, *Paragloborotalia siakensis*, *D. altispira* gr., *Turborotalita* cf. *quinqueloba*, *Praeorbulina glomerosa circularis*, ***Orbulina suturalis***	MMi5a	
BX8	1.6	poor	*Helicosphaera carteri (C), Sphenolithus heteromorphus (C), S. moriformis (C), H.euphratis (R), H. mediterranea (R)*	MNN5a	very little residue made up of sediment fragments, quartz, plant remains. Planktonic foraminifera rare, preservation good.	*Globigerina praebulloides*, *Globorotalia praescitula*, *G. trilobus*, *Dentoglobigerina altispira* gr., *Paragloborotalia siakensis*, *Globoturborotalita woodi*, *G. sicanus*, *Praeorbulina* cf. *glomerosa curva*, *Praeorbulina glomerosa glomerosa*, *Praeorbulina glomerosa circularis*, ***Orbulina suturalis***	MMi5a	
BX6	1.25	BARREN			little residue, terrigenous fraction very abundant (sediment fragments,quartz, mica), very abundant plant remains, planktonic foraminifera rare, preservation moderate	*Globigerinoides trilobus*, *Dentiglobigerina altispira* gr., *Globigerina praebulloides*, *Globorotalia praescitula*, *Turborotalita* cf. *T. quinqueloba*, *Paragloborotalia siakensis*, *Praeorbulina* spp. (the apertures are not always visible), ***Orbulina suturalis***	MMi5a	
BX5	1	UNDEFINABLE						
BX4	0.75	BARREN			elongated crystals (A), oxydized sediments fragments (A), plant remains.			barren
BX3	0.5	poor	*Helicosphaera carteri (C), S. moriformis (A), H. ampliaperta (R), H. euphratis (R/C), H. mediterranea (R/C), H. intermedia (R/C)*	MNN4c				
BX2	0.25	poor	*Helicosphaera carteri (C), Sphenolithus heteromorphus (C/R), S. moriformis (C), H.euphratis (R), H. mediterranea (R)*	MNN4b	sediment fragments, elongated crystals, mica (muscovite), oxydized and pyritized fragments, plant remains.			barren
BX1	0	poor	*Helicosphaera carteri (C), Sphenolithus heteromorphus (C/R), S. moriformis (C), H.euphratis (R), H. mediterranea(R)*	MNN4b	very little residue made up of pyritized fragments, pyrite, sediment fragments, plant remains. Foraminifera in trace, preservation moderate/poor (recrystallized and deformed)	*Globigerinoides trilobus, G.* cf. *sicanus*, ***Praeorbulina glomerosa glomerosa***, ***Praeorbulina glomerosa circularis?***	MMi4c (/d?)	nearly barren

In the Brebu section, preservation is generally poor and calcareous nannofossil assemblages are often poorly preserved in the analyzed samples. However, in each sample the most relevant biostratigraphic markers were individuated, allowing to ascribe the deposits from the base to the top to the early Langhian MNN4b Subzone (BX1 and BX2 at 0 and 0.25 m), to the middle Langhian MNN5a Subzone (BX3 to BX10: 0.5–2 m), and to the late Langhian MNN5b Subzone (BX13, 2.4 m) (Table 1, Fig. 7).

The preservation of the calcareous nannofossil assemblages in the Dracului Valley (DV) section is very poor in the low-intermediate portion of the sampled interval, between samples DV01 and DV17 (base of section to 21.8 m). Conversely, starting from sample DV19a (25.7 m), a good degree of preservation allows ascribing the deposits to the middle to late Langhian MNN5a (samples DV19a-DV21; 25.7–30.7 m) – MNN5b (sample DV22; 33 m) subzones. Sediments from Ciceu-Giurgesţi Section are in general well-preserved. The bio-chronostratigraphic analysis allowed ascribing the samples between the early Langhian MNN5b Subzone (samples CG01-CG11; 6.5–33 m) and the late Langhian to early Serravallian MNN5c Subzone (samples CG12-CG24; 36.4–80 m). In the Cepari section, the preservation degree is very poor in the lowermost samples (CP01-CP3B; 0.1–3.4 m), and good in the upper part of the section (samples CP7b-CP10; 14.7–18.7 m). The bio-chronostratigraphic analysis allowed framing the deposition in the middle Langhian MNN5a Subzone (Fig. 7).

#### 2.3.2 Planktonic foraminifera

Planktonic foraminifera from the lower part of the Campiniţa section (up to sample CA08 at about 139 m, Doftana Fm) are generally absent or very diluted (Table 1). In a few stratigraphic levels (CA30 and CA29 at about 87 m) ill-preserved double-keeled planktonic specimens are evidence of reworking of Cretaceous sediments. Miocene planktonic foraminiferal assemblages are represented by rare and scattered occurrences of *Dentoglobigerina* spp*., Globigerina praebulloides, Globigerinella* spp*., Globigerinoides trilobus* and *Paragloborotalia siakensis*. In only two samples (CA44 at about 62 m and CA19 at about 102 m) a single occurrence of *Praeorbulina glomerosa glomerosa* has been found suggesting the attribution to Subzone MMi4c (Fig. 7). However, the biostratigraphic interpretation is weak because of the extreme rarity of planktonic foraminifera. In a few samples (CA12, CA11, CA10 and CA9B, ∼118–134 m) occurrences of *Streptochilus* spp.*/Bolivin* spp. have been recorded in the >63 < 125 μm fraction of the washed residue (Table 1, Fig. 6).

**Fig. 5:**
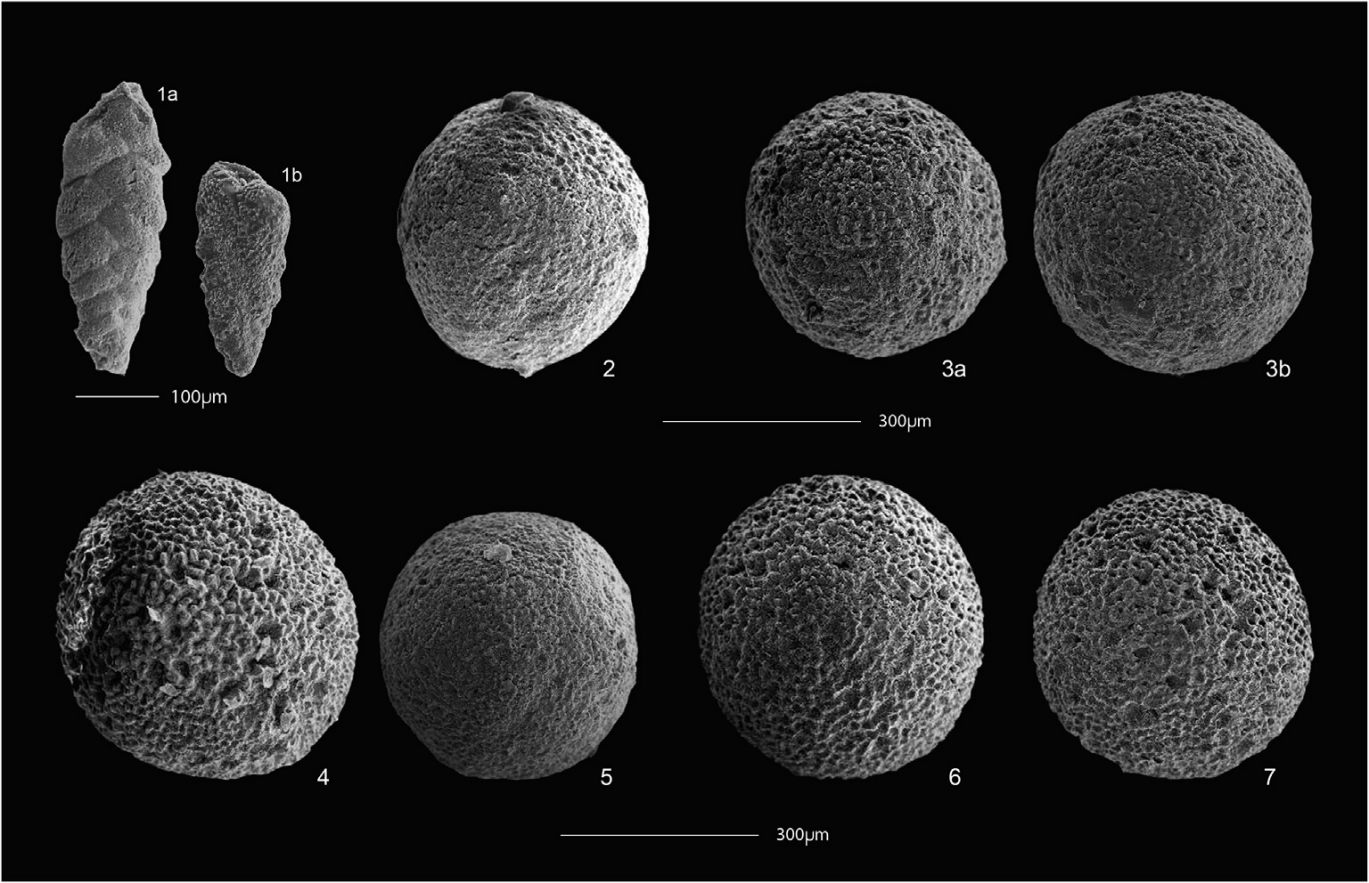
**Planktonic foraminifera**. (1a, b) *Streptochilus* sp.*/Bolivinina* spp. (sample CA10); (2) *Praeorbulina glomerosa glomerosa* (sample CF6); (3a, b) *P. glomerosa circularis* (sample CF6); (4) *O. suturalis* (sample CA7); (5) *O. suturalis* (sample CF6); (6) *Orbulina* intermed. *suturalis-universa* (sample CA7); (7) *O. universa* (sample CA7).

Samples from the uppermost part of the section (from 139 m up to the top, Campiniţa Fm) generally yield abundant planktonic foraminifera mainly represented by globigerinids (in some levels), *Globigerinoides trilobus, Globigerinoides* cf*. G. sicanus, Paragloborotalia siakensis, Globorotalia praescitula, Globorotalia* spp., *Praeorbulina glomerosa glomerosa, P. glomerosa circularis* and *Orbulina suturalis* (Fig. 5). The occurrence of *O. suturalis* allows the attribution of the interval including samples CX13 to CA5 (139.3–147.4 m) to Subzone MMi5a. In sample CA07 (at about 150 m) the occurrence of *Orbulina universa* indicates Subzone MMi5b (Fig. 7).

**Fig. 6:**
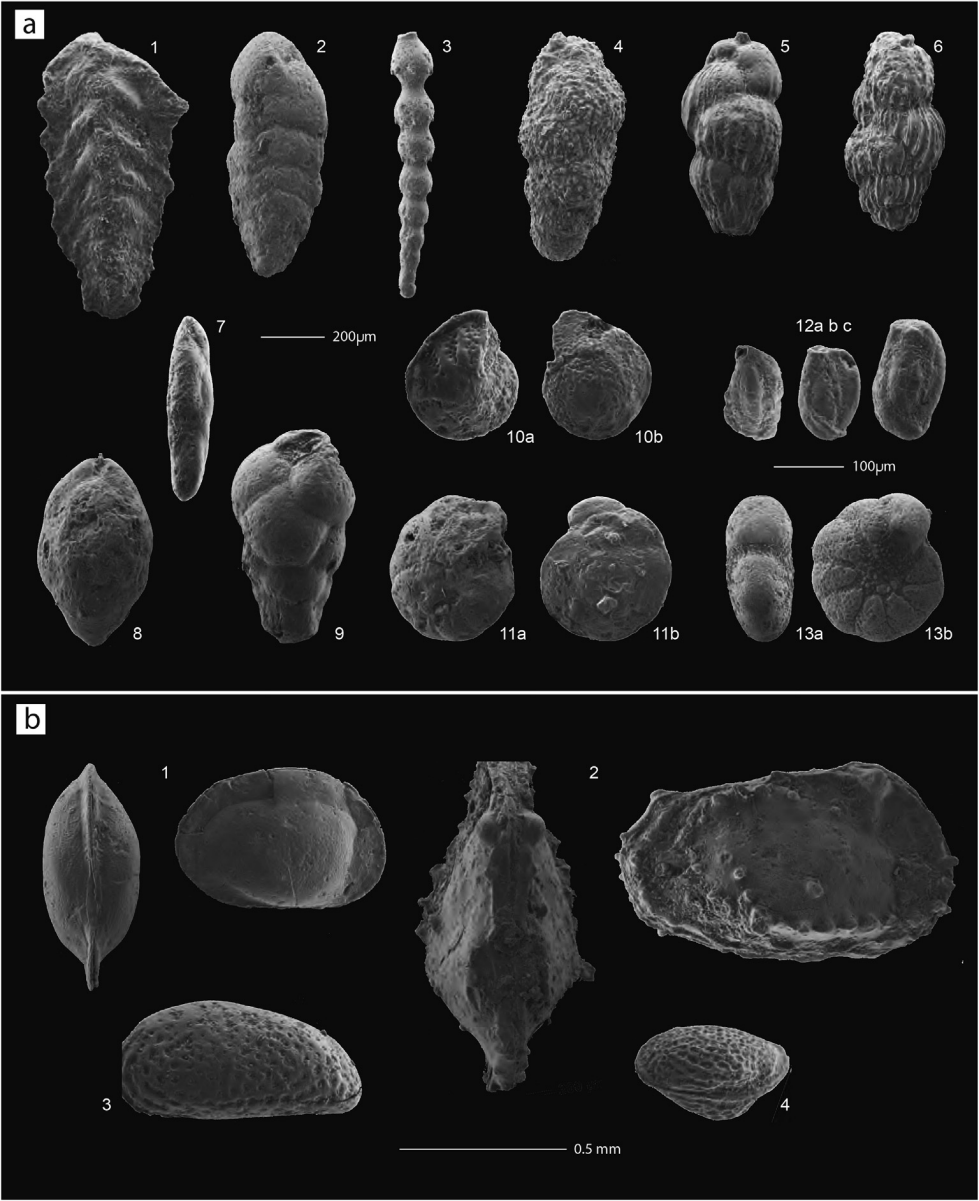
a. **Ostracods**. (1) *Loxoconcha punctatella* (sample CA8); (2) *Pterygocythereis calcarata* (sample CP2); (3) *Cytheridea acuminata* (sample CP2); (4) *Loxoconcha kochi* (sample CP2). b. **Benthic foraminifera**. (1) *Spiroplectinella carinata* (sample CG14); (2) *S. deperdita* (sample CP2); (3) *Stilostomella* sp. (sample CP2); (4) *Uvigerina* cf. *U. acuminata* (sample CG14); (5) *Uvigerina semiornata* (sample CG14); (6) *Uvigerina* cf. *U. venusta* (sample CG14); (7) *Fursenkoina acuta* (sample CP2); (8) *Globobulimina* sp. (sample CP2); (9) *Bulimina elongata* (sample CG14); (10) *Cibicides* cf. *C. ungerianus* (sample CG14); (11) *Cibicides dutemplei* (sample CG17); (12) miliolid spp. (sample CA8); (13) *Porosononion granosum* (sample CP2).

**Fig. 7:**
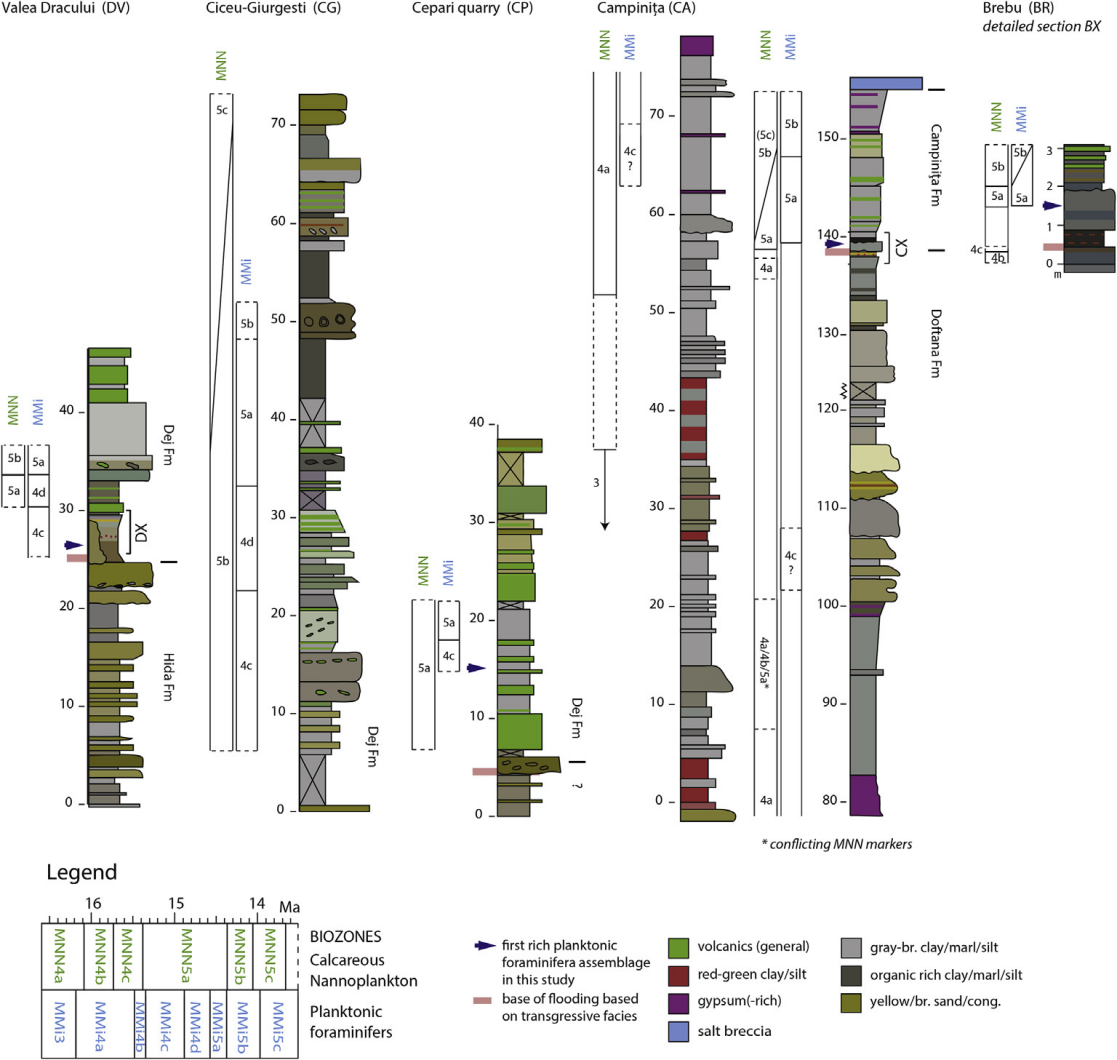
**Biozones of studied sections.** Simplified stratigraphic logs of the sections with the boundaries of the biozones indicated. For Brebu only the part of the section with biostratigraphic data is depicted. See Fig. 2 for stratigraphic details of the sections and sample levels for biostratigraphy. See text for determination of the biozones.

In the Brebu section, most of the samples are barren in fossils or contain very rare planktonic foraminifera. Only the uppermost samples (BX10 and BX13) yield abundant and moderately preserved planktonic foraminifera. The most recurrent taxa are *Dentoglobigerina altispira* gr.*, Globigerina praebulloides, Globigerinoides sicanus, Globigerinoides trilobus, Globorotalia praescitula, Globoturborotalita woodi, Paragloborotalia siakensis, Praeorbulina glomerosa* s.l. and *Orbulina suturalis*. Rare specimens of *Praeorbulina glomerosa glomerosa* and *P.* cf. *glomerosa circularis* occur in the lowermost sample (BX1) suggesting Subzone MMi4c/d?, while *Orbulina suturalis* has been found in the sample interval between BX06 and BX13 allowing the attribution of this interval to Subzone MMi5a. In the latter sample few specimens of *O. suturalis* show more evolved characters close to *O. universa*, whose first occurrence identifies the base of Subzone MMi5b (Table 1, Fig. 7).

Samples from the lower part of the Valea Dracului section, up to 25 m, are generally barren or nearly barren in foraminifera. In sample DV 17 (at ∼22.8 m) a single specimen of *Praeorbulina glomerosa glomerosa* has been found suggesting Subzone MMi4c? Note, however, that the reliability of this biostratigraphic attribution is weak since planktonic foraminifera are extremely rare in this sample. Samples from the upper part of the section, from 25 m to the top, contain more abundant planktonic foraminiferal assemblages, mainly represented by *Dentoglobigerina altispira* gr.*, Globigerinoides sicanus, Globigerinoides trilobus, Praeorbulina glomerosa* s.l. and *Orbulina suturalis*. Specifically, the occurrence of *Praeorbulina glomerosa circularis* in samples DV20 and DV21, and the occurrence of *O. suturalis* in sample DV22, indicates Subzones MMi4d and MMi5a, respectively (Table 1, Fig. 7).

Planktonic foraminiferal assemblages in the Ciceu-Giurgesţi section are moderately to poorly preserved and are mainly represented by *Dentoglobigerina* spp., *Globigerina praebulloides*, *Globigerinella* spp. *Globigerinoides sicanus*, *Globigerinoides trilobus*, *Globorotalia scitula*, *Praeorbulina glomerosa* s.l. and *Orbulina suturalis*. The lowermost part of the section (sample CG01) is tentatively assigned to Subzone MMi4c? On the basis of an uncertain single occurrence of *Praeorbulina glomerosa glomerosa*, while the occurrence of *Praeorbulina glomerosa circularis* in sample CG08 is indicative of the Subzone MMi4d (Fig. 7). The middle part of the section (samples CG11, CG12 and CG14: 33–47.5 m) can be assigned to Subzone MMi5a because of the presence of *Orbulina suturalis*. Note, however, that preservation of specimens of the *Praeorbulina*/*Orbulina* group is poor complicating the classification to a specific biozone. The absence of biostratigraphic marker species hampers a precise biostratigraphic interpretation of the upper part of the section (above 47.5 m: Table 1).

Finally, the lower part of the Cepari section (up to about 5 m) is characterized by very rare to common poorly preserved planktonic foraminifera, mainly represented by *Globigerina praebulloides*, *Globigerinoides* cf. *G. sicanus*, *Globigerinoides trilobus* and *Paragloborotalia siakensis*. The absence of marker species does not allow a precise biozonal assignment of this interval. Upward, planktonic foraminiferal assemblages are characterized by the occurrence of *Praeorbulina* and *Orbulina* spp., but their poor preservation generally hampers the classification at specific level. However, sample CP7B (14.7 m) is characterized by the presence of *Praeorbulina glomerosa curva* and *P. glomerosa glomerosa* indicating Subzone MMi4c, and sample CP10 (18.7 m) contains *P. glomerosa glomerosa*, *P. glomerosa circularis* and few specimens close to *Orbulina* (*O.* cf. *suturalis*) indicating Subzone MMi4d/MMi5a(?) (Table 1, Fig. 5, Fig. 7).

#### 2.3.3 Benthic foraminifera and ostracods

In the samples containing benthic foraminifera, the assemblages vary from extremely poor to relatively rich (Table 1). In the SE Carpathian Foredeep, in the Campiniţa (CA) section, the interval below the open marine flooding (117.9–136.5 m; samples CA13, CA10 and CA9) is quartz-rich and the relatively rare hyaline taxa are poorly preserved. Grain size sorting suggests transport or winnowing. Plant remains in CA10 and CA13 indicate a near-coastal environment, possibly brackish and in the vicinity of a river plume. Most taxa in these samples are restricted to the inner-mid shelf; some taxa (miliolids, *Hanzawaia boueana*, *Rosalina globularis*) tolerate a certain degree of hypo- and hypersalinity. In sample CA09, preservation of fish remains suggests oxygen restriction (since phosphate is preserved under anoxic conditions), and a specimen of the planktonic foraminifer *Morozovella* sp. indicates reworking of Paleogene sediments. Sample CA08, located in the base of the flooding at 138.7 m, mainly contains diverse miliolids together with numerous, nearly monospecific smooth ostracods (*Loxoconcha punctatella*) pointing to an oxic, shallow-marine environment with deviating salinity (Table 1; Fig. 5).

In the Transylvanian Basin, the washed residue of the sample collected from the basal Dej Fm just above the flooding in the Valea Dracului (DV) section (DV19A; 25.7 m) is large, well-sorted and clean (mainly quartz, lithic fragments), suggesting a high-energy environment, possibly a beach. Absence of planktonic foraminifera and rare, recrystallized benthic foraminifera: miliolid, *Elphidium* sp. and *Textularia* sp. suggest shallow-marine waters. Higher up, the washed residue of sample DV19 (29.5 m) contains only two hyaline specimens (*Cibicides* -*lobatulus* and a *C.* cf. *ungerianus*?) which may be displaced or reworked. Planktonic foraminifera are relatively common and minor pyrite and fish remains are present. Together with the virtual absence of benthic foraminifera this might point to bottom-water oxygen deficiency in shelf waters of normal marine salinity.

In the Ciceu-Giurgesţi (CG) section, two samples in the Dej Fm at 47.5 (CG14) and 55.4 m (CG17) contain comparatively rich and diverse benthic foraminiferal associations (Fig. 5). Most specimens are large, although some more fragile taxa are also present. The samples contain a mix of shallow-water (*Ammonia tepida, Discanomalina coronata, Elphidium* spp.*, Rosalina bradyi*, discorbids) and deeper-water, mid (-outer) shelf taxa (*Cibicides dutemplei, C. ungerianus,* large *Uvigerina* spp.: *Uvigerina* cf. *U. acuminata*, *Uvigerina* cf. *U. venusta*, *U. semiornata*), indicating a mid-shelf environment for sample CG17. Sample CG14 may have been deposited in slightly deeper water (outer shelf depth) since it also contains *Globocassidulina subglobosa*, *Pullenia bulloides*, *Sphaeroidina bulloides* and nodosariids. The sediments were possibly deposited in slightly dysoxic environments, indicated by a relatively high percentage of species thriving under limited oxygenation and/or high organic load (*Bolivina spathulata, Bulimina aculeata, B. elongata, Fursenkoina acuta, Globobulimina* sp.*, Uvigerina* cf. *U. acuminata, U. continuosa, U. semiornata*). Bimodal grain size sorting, especially in CG17 might indicate transport. This sample contains large, reworked miliolids and part of the shallow-water foraminifera and the (scarce) ostracods are pyritized, and may be reworked too.

Sample CP02, collected in the Cepari (CP) section 3 m below the flooding surface contains relatively abundant, often large Miocene benthic foraminifera (Fig. 6). Bimodal grain size sorting suggests transport; taxa normally occurring on the shallow shelf (*Ammonia beccarii*, *A. tepida, Discanomalina coronata, Elphidium* spp.) may have been transported to mid-shelf depths (indicated by (scarce) *Cibicides* spp*., Cassidulina carinata, Gavelinopsis lobatula, Hanzawaia boueana, Spiroplectinella deperdita*). Large, pyritized miliolids are almost certainly reworked. Common *Bulimina elongata*, *Globobulimina* spp. and *Fursenkoina acuta*, together with pyrite might point to a high organic load and associated oxygen limitation, although burrows confirm the presence of some oxygen. Both smooth (*Loxoconcha punctatella*) and ornamented ostracods occur (*Cytheridea acuminata, Loxoconcha kochi, Pterygocythereis calcarata*; Fig. 6).
