# Supplementary material for: Litho- and biostratigraphic data of lower-middle Miocene sections in the Transylvanian basin and SE Carpathian Foredeep (Romania)
Source: Data Brief. 2019 Apr 17;24:103904. doi: 10.1016/j.dib.2019.103904 (PMC6525294; doi:10.1016/j.dib.2019.103904)
Supplement: Supplementary file 1 — Multimedia Component 1 [file mmc1.doc]

# CONFLICT OF INTEREST STATEMENT

***Data in Brief***

Manuscript Title: “Litho- and biostratigraphic data of lower-middle Miocene sections in the Transylvanian basin and SE Carpathian Foredeep (Romania)”

1. Royalties from a company or supplier (The following conflicts were disclosed)

No

2. Speakers bureau/paid presentations for a company or supplier (The following conflicts were disclosed)

No

3A. Paid employee for a company or supplier (The following conflicts were disclosed)

No

3B. Paid consultant for a company or supplier (The following conflicts were disclosed)

No

3C. Unpaid consultants for a company or supplier (The following conflicts were disclosed)

No

4. Stock or stock options in a company or supplier (The following conflicts were disclosed)

No

5. Research support from a company or supplier as a Principal Investigator (The following conflicts were disclosed)

No

6. Other financial or material support from a company or supplier (The following conflicts were disclosed)

No

7. Royalties, financial or material support from publishers (The following conflicts were disclosed)

No

8. Medical/Orthopaedic publications editorial/governing board (The following conflicts were disclosed)

No

9. Board member/committee appointments for a society (The following conflicts were disclosed)

No


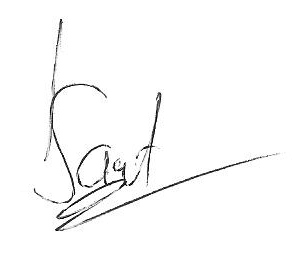
Author Name : Karin Sant Author Signature Date: Jan 31 2019
